# Metagenomics revealed a correlation of gut phageome with autism spectrum disorder

**DOI:** 10.1186/s13099-023-00561-0

**Published:** 2023-08-04

**Authors:** Khashayar Shahin, Abbas Soleimani-Delfan, Zihan He, Philippe Sansonetti, Jean-Marc Collard

**Affiliations:** 1 Center for Microbes, Development, and Health (CMDH), Institute Pasteur of Shanghai/Chinese Academy of Sciences, Life Science Research Building, 320 Yueyang Road, Xuhui District, Shanghai, 200031 China; 2 Department of Cell and Molecular Biology & Microbiology, Faculty of Biological Science and Technology, University of Isfahan, Hezar Jereeb Street, Isfahan, 81746-73441 Iran; 3 Present Address: Enteric Bacterial Pathogens Unit & French National Reference Center for Escherichia Coli, Shigella and Salmonella, Department of Global Health, Institut Pasteur, 28 rue du Dr Roux, 75724 Paris cedex 15, France

**Keywords:** Autism spectrum disorders, Gut phageome, crAssphage, Metagenomics

## Abstract

The human gut bacteriome is believed to have pivotal influences on human health and disease while the particular roles associated with the gut phageome have not been fully characterized yet with few exceptions. It is argued that gut microbiota can have a potential role in autism spectrum disorders (ASD). The public microbiota database of ASD and typically developing (TD) Chinese individuals were analyzed for phage protein-coding units (pPCU) to find any link between the phageome and ASD. The gut phageome of ASD individuals showed a wider diversity and higher abundance compared to TD individuals. The ASD phageome was associated with a significant expansion of *Caudoviricetes* bacteriophages. Phages infecting *Bacteroidaceae* and prophages encoded within *Faecalibacterium* were more frequent in ASD than in TD individuals. The expansion and diversification of ASD phageome can influence the bacterial homeostasis by imposing pressure on the bacterial communities. In conclusion, the differences of phages community in in ASD and TD can be used as potential diagnosis biomarkers of ASD. Further investigations are needed to verify the role of gut phage communities in the pathogenesis of ASD.

**Supplementary Information:**

The online version contains supplementary material available at 10.1186/s13099-023-00561-0.

## Introduction

The most abundant microorganisms in our biosphere are phages (viruses) that have special roles in the regulation of microbial communities [1]. The ecological functions of phages and their correlation with their host cells in bacterial communities remain often unclear [2]. Although phages have been extensively utilized for therapeutic and biotechnology purposes, the investigation of natural phage communities in the gut is relatively new in microbial diversity [2]. In recent years, changes in bacteriophages community, phageome, and their direct or indirect modulations on the gut microbiome were investigated in some diseases and disorders in humans [3]. Bacteriophage population changes in the human intestine has a strong correlation with the occurrence of diseases [4]. For example, patients suffering from Parkinson's disease had a higher frequency of lytic lactococcal phages, which was in agreement with the observed declining of lactic acid bacteria responsible for dopamine production [3]. It was reported in stunted patients the bacteriophages mediated shift in the gut bacteriome resulted in digestion and/or absorption disorders that eventually led to stunting [4]. Both obesity as another burden of malnutrition [5] and its potential consequence, type 2 diabetes mellitus (T2DM), are linked to gut microbiota dysbiosis with the role of bacteriophages to be understood [6]. It was reported obesity with T2DM (ObT2) has a stronger impact on the diversity and abundance of gut phageome than Ob-non-T2 [7]. The correlation of *Streptococcus* phages and its bacterial hosts are reduced in ObT2, indicating ObT2 may aggravate the obesity-related phages signatures, implying the significance of the gut phageome to the development of obesity and T2DM [7].

Prophages (lysogenic phages) are highly abundant in bacterial genomes (> 80%), hence are important players affecting bacterial diversity, metabolism, and function of a microbiota, and consequently the hosting biotic or abiotic habitats such as a human being [8]. Nevertheless, the potential role of phageome on the relationship between bacteriome and diseases has been poorly studied. Autism spectrum disorder (ASD) is a neurologic disorder with an occurrence rate of 1 in 160 children worldwide [9]. The critical roles of various factors including heredity, diet, pollution and recently the gut microbiota in ASD has been studied [10]. Dan et al. [11] on 30 children with ASD signs and 30 non-ASD (TD) individuals as the controls indicated that ASD individuals’ gut microbiota was significantly changed, mainly by a decreased diversity with depletion of *Sutterella*, *Prevotella* and *Bacteroides* species, accompanied by dysregulation of associated metabolic activities, yet they did not address the abundance of phages as the modulator of the microbiome.

This study aims to shed light on gut phageome structure and its potential role in ASD, based on the metagenomics raw data obtained by Dan et al. [11], the phage abundance in healthy and ASD individuals was determined and phage variation was individually and collectively analyzed.

## Materials and methods

### Information of the cohort study

The metagenomics data is from Dan et al. [11] cohort study from May 2016 to August 2017. Briefly, 143 cases of ASD children (2–13 years old, 130 male, 13 female, 52 constipated symptoms, and 5 diarrhea symptoms) and 143 cases of TD (2–13 years old, 127 male, and 16 female) as the control group of children matched on age and sex were recruited to the cohort study. ASD individuals had been all diagnosed according to the Diagnostic and Statistical Manual of Mental Disorders, 5th Edition [12]. The majority of cases in the groups were males as autism is more frequently diagnosed in male individuals [12].

The stool samples of all 143 in each study groups have been used for DNA extraction and 16S rRNA sequencing but only 30 ASD (3–13 years old, 27 male and 3 female, 30 constipated symptoms) and 30 aged-matched TD (3–11 years old, 28 male and 2 female, 0 constipated symptoms) were selected for future metagenomics analysis (Additional file 1). The metagenomics data had been retrieved from sequencing of total DNA extracted from feces samples (no viral DNA extracting method used) using Illumina Hiseq X platform (insert size 350 bp, read length 150 bp), which could undoubtedly influence the quality and quantity of the extracted viral DNA. The lack of specific viral DNA and RNA extraction kit(s) could restrict the metagenomics to double-strand DNA contigs and missed the single-strand DNA (ssDNA) and RNA phages fragments in the phage pool. The raw data of whole human gut metagenomes of all available 60 samples which could be considered as the only available sequences in ASD cases (accession numbers SRR7057620 to SRR7057679) have been deposited to GEO under accession number GSE113540.

### Metagenomics analysis

**Fig. 1:**
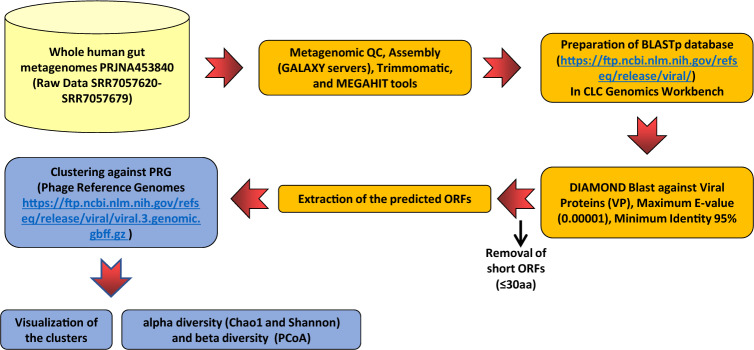
Schematic workflow for metagenomic analysis and phage taxonomic classification in ASD and TD individuals

The whole human gut metagenomes raw data were obtained from ENA servers (https://www.ebi.ac.uk/ena/browser/view/PRJNA451479?show=reads) and then transferred to galaxy server individually (https://usegalaxy.org/). Each set of data was assembled through a de novo assembling algorithm in MEGAHIT (https://toolshed.g2.bx.psu.edu/repository?repository_id=2f02857913c9a24f) for metagenomics assembly by minimum multiplicity for filtering (k_min + 1)-mers 2. The minimum contigs output length was 200 following Trimmomatic operation with the preset setting for data quality control. The assembled contigs from 30 ASD and 30 TD individuals were imported to CLC genomics workbench V20 (CLC-GW v20). For analyzing the phage coding regions inside the contigs, a database based on phage protein-coding units (pPCU) was prepared (https://ftp.ncbi.nlm.nih.gov/refseq/release/viral/) until 29/5/2023. The DIAMOND BLASTx [13] and annotation were conducted through default settings in CLC-GW v20 (genetic code 11, maximum E-value 0.00001, minimum identity 95%, and minimum reference sequences coverage 0% with standard search). Briefly, the megahit results were aligned against the prepared database based on all viral sequences and third part annotation (TPA) data that have been already deposited and available in GenBank (The database is available upon request). Moreover, the results were normalized based on the default setting on CLC-GW v20. For an accurate phage taxonomic profile of the open reading frames (ORFs), ORFs smaller than 30 amino acids were removed. Next, viral open reading frames (vORFs) that were identified by DIAMOND BLASTx [13] were extracted and subjected to taxonomic profiling. Similar phages were clustered in three taxonomic levels: family, genus, and species using reference-based OTU clustering by CLC-GW v20 against the database. Extracted phage contigs were clustered into OTUs by multiple alignments using MUSCLE and finally one contig was chosen for taxonomic analysis against the database that was previously downloaded (RefSeq genomes: https://ftp.ncbi.nlm.nih.gov/refseq/release/viral/) with taxonomic similarity 80% and similarity percentage 95%, minimum occurrence 0%, and other default settings in CLC-GW v20 (Fig. 1).

VirSorter (v 1.0.3) was used for identifying phage contigs and abundancy [14]. Then CLC-GW (v20) and DIAMOND were used to identify the viral ORFs based on the default setting. The results of this analysis were compared with the results of the developed pipeline in the present study to confirm the accuracy and coverage.

Alpha and beta diversity were calculated with R package phyloseq and vegan (R Foundation for Statistical Computing). Data processing and visualization were performed by R packages dplyr, readr, stringr, ggplot2, aPCoA, pheatmap, and ggsignif. Two-tailed Wilcoxon’s rank sum test was used to determine statistically significant differences for alpha diversity indices between 2 groups and a P-value of < 0.05 was considered statistically significant.

## Results and discussion

### Autism Spectrum Disorder Contributed to Gut Phageome Alterations.

Using DIAMOND BLASTx, 78,585 and 74,228 contigs were found in ASD and TD groups including complete and incomplete phage domain protein and amino acids, respectively (the data are available upon request). Using Refseq, 1878 and 4774 contigs were identified as phages in ASD and TD groups, respectively (Additional file 2). VirSorter analysis identified 16,772 contigs in ASD and 17,632 contigs in TD in three categories while using Refseq a number of 808 and 1237 contigs were considered as hallmark phages in ASD and TD groups, respectively (Additional file 3), a significantly smaller number of contigs compared to the developed pipeline in the present study. All VirSorter identified contigs were also covered by our pipeline (Additional file 4). The results indicated that in both reference phage (Refseq) and other phage genus (TPA and non-classified phages) are more dominant than the results obtained with other pipelines (at this point Virsorter). The identified functional while ORFs are presented in Additional file 5. The ssDNA phages, *Microviridae* and *Inoviridae* for instance, were not identified in neither in ASD nor TD groups. This could be due to two factors, the abundance of ssDNA phages in the gut system [15] and not using viral-specific genome extraction method and library preparation by Dan et al., [11]. The positive associations between fecal dsDNA phages (order *Caudovirales*) and parameters of the brains’ executive functions have been discussed elsewhere [16]. Therefore, it can be hypothesized that the observed changes in gut phageome could be a potential biomarker for some of the brain performance and behaviors [8].

**Fig. 2:**
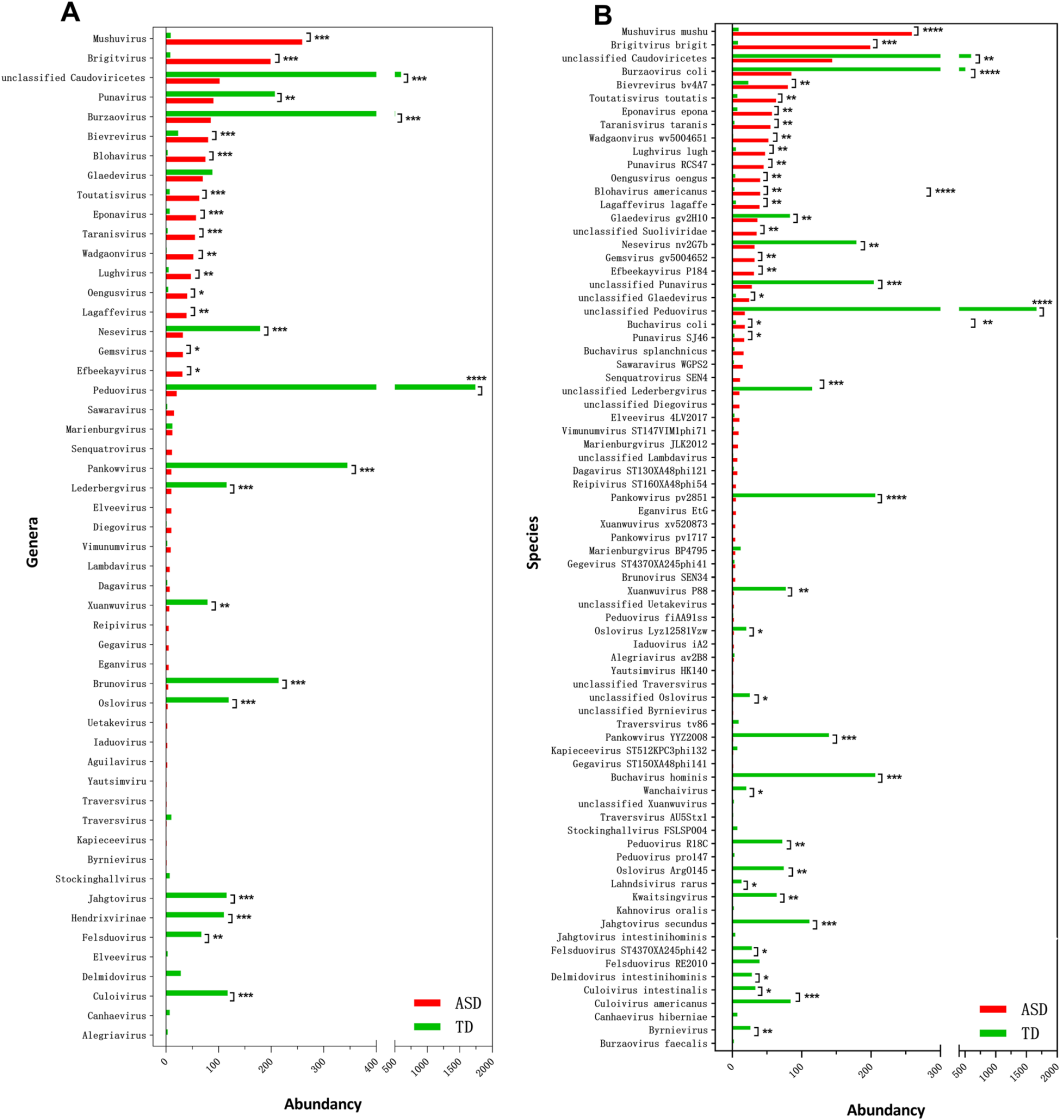
Taxonomic distribution of gut phageome in ASD and TD. **A** The differential abundance of phage genera detected in ASD and TD. **B** The differential abundance of different phage species identified in ASD and TD. * P < 0.05, ** P < 0.01, *** P < 0.001 and **** P < 0.0001 showed a significant difference of the particular phage in ASD with TD. Please refer to Additional File 2 for the detail of differential abundance of the phages

Mayneris-Perxachs et al. [8] reported correlations between *Siphoviridae* family and a better executive function and memory in mice. Transplantation of a microbiome enriched with a high level of *Siphoviridae* phages (> 90%) in mice promoted objects recognition and up-regulated memory-promoting immediate early genes in the prefrontal cortex [8]. On one hand, children affected by different levels of ASD have a poor executive function and memory [17]; and on the other hand the different abundancy of phages in TD and ASD individuals which is observed in the current study, could be a contributing factor of such symptom (Fig. 2A). Hence, there could be link between the prevalence of different genera of phages and brain function. Although the precise roles of phages genera in the microbiome-brain axis are poorly understood [8], their different abundance in ASD compared to TD individuals may result in an enhancement of ASD. Induction of prophage from commensal bacteriome or obtaining new phages from environments e.g., food and direct contact with community might explain the increased level of *Caudoviricetes* phages [18].

A deeper analysis based on conserved sequences of bacteriophages like phage DNA polymerase and terminase large subunits revealed the presence of enriched phage genera in ASD compared to TD (Fig. 2A). Among the different genera, *Mushuvirus* (P < 0.001), *Brigitvirus* (P < 0.001), *Toutatisvirus* (P < 0.001), *Eponavirus* (P < 0.001), *Taranisvirus* (P < 0.001), *Wadgaonvirus* (P < 0.01), *Lughvirus* (P < 0.01), *Oengusvirus* (P < 0.05), *Lagaffevirus* (P < 0.01), *Gemsvirus* (P < 0.05), and *Efbeekayvirus* (P < 0.05) were more abundant in ASD while *Punavirus* (P < 0.01), *Burzaovirus* (P < 0.001), *Nesevirus* (P < 0.001), *Peduovirus* (P < 0.0001), *Pankowvirus* (P < 0.001), *Lederbergvirus* (P < 0.001), *Brunovirus* (P < 0.001), *Oslovirus* (P < 0.001), *Jahgtovirus* (P < 0.001), *Hendrixvirinae* (P < 0.001), *Felsduovirus* (P < 0.01), *Culoivirus* (P < 0.001), and *Delmidovirus* (P < 0.01) were more abundant in TD (Fig. 2A).

Among the different viral species, *Faecalibacterium* phages (FP) including Toutatisvirus toutatis (P < 0.01), Mushuvirus mushu (P < 0.0001), Brigitvirus brigit (P < 0.001), Taranisvirus taranis (P < 0. 01), Eponavirus epona (P < 0. 01), Oengusvirus oengus (P < 0. 01), Lagaffevirus lagaffe (P < 0. 01), and Lughvirus lugh (P < 0. 01) had the largest relative abundance in ASD compared to TD (Fig. 2B). The bacterial hosts of these phages, *Faecalibacterium* spp., mainly represented by *Faecalibacterium prausnitzii,* are highly presented in the human gut microbiota (5–15% of the human gut microbiome). Those bacteria produce butyrate and other beneficial substances for human health through mechanism such as anti-inflammatory effects or maintaining the Th17/Treg balance [19]. A correlation was observed between the depletion of *Faecalibacterium* and Crohn’s disease [20], obesity in infants [21] type II, diabetes [22] and aging [23].

In the present study, a high rate of *Faecalibacterium* phages was observed in ASD compared to TD individuals pointing out a possible role of *Faecalibacterium* and their prophages in autism. The higher frequency of eight *Faecalibacterium* phages in ASD individuals could change either the level of *Faecalibacterium* in the gut (via possible prophage induction and the start of the lytic cycle) or impact the metabolic activities and the function of *Faecalibacterium* spp. in gut microbiota, or both. The higher abundance of *Faecalibacterium* spp. in ASD compared to TD individuals, rather than a depletion [11, 24, 25] suggests an impact on metabolic activities instead of the induction of the lytic phase inducing depletion of *Faecalibacterium* spp. as observed by Cornuault et al. [19], in patients suffering from an inflammatory bowel disease (IBD). The possible roles of prophages on bacterial metabolism mediated by auxiliary metabolic genes (AMGs) were highlighted for some bacteria. For example, a significant increase in middle-chain fatty acids (MCFAs) such as hexanoic acid was observed by Dan et al. [11] in the ASD group. Hexanoic acid can be produced by members of the *Clostridium* cluster IV and *Ruminococcaceae* bacterium CPB6 [26]. The *Oscillospiraceae* family including *Faecalibacterium* sp. CAG: 74, *Subdoligranulum variabile*, *Clostridium* sp. CAG: 269 and *Eubacterium* sp. CAG: 38 displayed a positive correlation with hexanoic acid level [11]. ASD individuals were associated with higher hexanoic acid levels in the blood in comparison to the TD group [27]. Further investigations are required to disclose the impacts of *Faecalibacterium* spp. prophages on host metabolism.

In our study, different crAssphages genera (belong to *Crassvirales* order) were identified in both ASD and TD individuals. For instance,

*Blohavirus* species (Buchavirus splanchnicus, Buchavirus coli, Blohavirus americanus, and.

Buchavirus hominis) in ASD were significantly abundant ((P < 0. 001) than TD while *Buchavirus* species (Buchavirus coli, Buchavirus hominis, Buchavirus splanchnicus, Burzaovirus coli and Burzaovirus faecalis) identified in TD were more abundant (P < 0.001) than ASD. Moreover, phages genera of *Canhaevirus* (Canhaevirus hiberniae), *Culoivirus* (Culoivirus americanus and Culoivirus intestinalis), *Delmidovirus* (Delmidovirus intestinihominis), and *Jahgtovirus* (Jahgtovirus intestinihominis and Jahgtovirus secundus) were identified only in TD indivituals (Fig. 2A and B). This observation was in parallel to the reported low abundance of *Bacteroides* spp. in ASD by Dan et al. [11]. Regarding the weak associations of *Bacteroides* with health or disease [28] and the overall high abundance of crAssphages in the human gut virome [29], it could be assumed that crAssphages diversity would be depended with their host (*Bacteroides* spp.) [29].

**Fig. 3:**
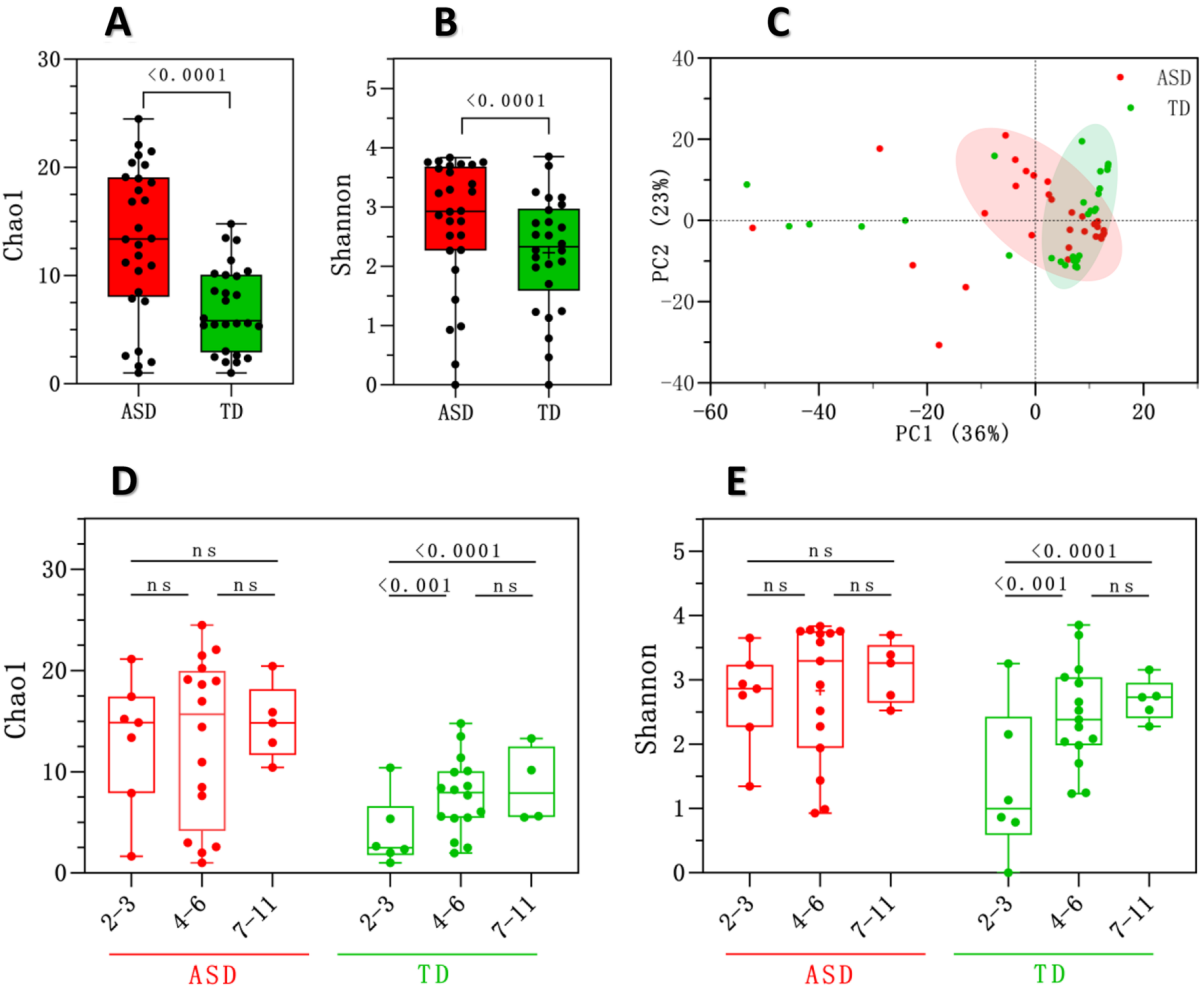
Changes in the gut phageome in ASD and TD individuals. **A** Chao1 richness and **B** Shannon’s diversity for the gut phageome of ASD and TD at the contig level. Kruskal–Wallis test, followed by Wilcoxon’s rank-sum test with Bonferroni’s correction were done for alpha diversity between the two test groups. **C** Principal coordinates analysis (PCoA) was performed based on the Bray–Curtis distance between individual viral groups. **D** Chao1 richness and **E** Shannon’s diversity of the gut phageome for TD and ASD according to age from 2 to 11

To investigate whether ASD individuals display a different gut phageome, a comparison of alpha diversity for phages between ASD and TD groups was performed as well. There was a significant difference between phage Chao1 richness and Shannon’s diversity of the ASD and TD groups (P < 0.0001, Fig. 3A, and P < 0.0001, Fig. 3B, respectively). Based on the alpha diversity, ASD individuals displayed unique gut phage profiles vis-à-vis TD (Fig. 3A and B). Additionally, principal coordinates analysis (PCoA) based on the Bray–Curtis distance between the cases revealed that the gut phageome structure of ASD was different from TD (Fig. 3C). The host-dependent factors such as age, sex, and gastrointestinal symptoms (constipation) were taken into consideration to analyze the phageome in ASD and TD individuals. As shown in Fig. 3D and E, the richness of phages enhanced in older TD individuals (mainly the 7–11 subgroup) compared to the youngest individuals (2–3 years age). However, the ASD subgroups showed no significant differences, neither in Chao1 richness nor in Shannon’s diversity. No additional analysis was performed for sex and gastrointestinal symptoms because the ASD group was composed of only 2 females and all cases suffered from severe constipation (Additional file 1). Dan et al., [11] reported a more heterogeneous and less diverse microbiome in ASD group, and different from the TD group [11]. They also noted that gut microbiota was relatively similar in all ASD age subgroups.

## Conclusion

The investigation of phage populations is still one of the main gaps in our understanding of human gut microbiome homeostasis or dysbiosis. Indeed, studies have highlighted the dysbiotic process only in bacterial communities, particularly in terms of autism. The present study is the first attempt to investigate the difference between the bacteriophages in ASD and TD individuals. The results indicated that *Caudoviricetes* is dominant in both groups. Among 124 phages identified in ASD and TD groups, *Faecalibacterium* phages are the most prevalent viruses in the ASD group. We also noted that while crAssphages were presence in both ASD and TD group they had different diversity.. In conclusion, however, it seems obvious that the gut phageome could play a role in the development of ASD individuals, a more comprehensive analysis and larger cohorts are required to better understand the role of the gut phageome in the pathogenesis of ASD.

### Supplementary Information

**Additional file 1.** Sample Selection Process for Metagenomics Analysis.

**Additional file 2.** Phage Contig Analysis in ASD and TD Groups.

**Additional file 3.** Viral Contig Classification in ASD and TD Groups.

**Additional file 4.** Coverage Validation of VirSorter Identified Contigs.

**Additional file 5.** Presentation of Identified Functional ORFs.

## Data Availability

The datasets and raw sequencing data used in this study (based on the personalized phage-based workflow) are available in the ENA servers (https://www.ebi.ac.uk/ena/browser/view/PRJNA451479?show=reads) under accession project number “PRJNA453840” (Raw Data SRR7057620- SRR7057679). Moreover, the pipeline and the database obtain using the developed pipeline in this study pipeline as well as VirSorter pipeline are all available upon request.
